# A Case of Uterine Myoma

**Published:** 1897-02

**Authors:** M. A. Crockett, R. F. Keyes

**Affiliations:** Gynecologist to the Erie County Hospital; Assistant Gynecologist to the Buffalo General Hospital; Clinical Lecturer in Gynecology, University of Buffalo


### Clinical Lecture.

### A CASE OF UTERINE MYOMA.

By M. A. CROCKETT, M. D.,

Gynecologist to the Erie County Hospital; Assistant Gynecologist to the Buffalo General Hospital; Clinical Lecturer in Gynecology, University of Buffalo.

Reported by R. F. KEYES, M. D.

THIS afternoon we shall consider the case of the patient upon whom several of you saw me operate last week. In connection with the clinical history I shall exhibit this specimen of uterine myoma, which was removed at the time of operation, and try to point out some of the relations between the symptoms and the pathologic condition.

It is of great importance for you to acquaint yourselves with the appearances and the "feel" of such a tumor, for you can then understand how it gives rise to certain signs upon vaginal and abdominal examination.

From our examination certain inferences were drawn. This specimen shows us how correct those inferences were. In other words, we have all the advantages of a post-mortem and cure the patient at the same time. The history of the patient is as follows :

Mrs. H., aged 50 ; married twenty years ; no pregnancies. About eight years ago there was discovered in the pelvis a tumor, giving rise to considerable pain and irritation of the bladder. At the hands of a distinguished specialist in another city she underwent a thorough course of electrical treatment, from which she derived marked benefit, but not complete restoration to health. Since the establishment of the menopause, one year ago, her symptoms have been quite severe, consisting of pains in the back of the neck, arms and legs, discomfort and throbbing in the region of the left groin, profuse leucorrhea, irritation of the bladder and constipation. In appearance the patient was stout, but very anemic.

*Examination.*—Heart and lungs normal. Urine, one pint in twenty-four hours. Specific gravity, 1028, acid ; no albumin nor sugar. Sediment, pus cells and urates.

*Abdominal Examination.*—No apparent distention on inspection. On palpation, a firm, irregular, slightly movable mass, about the size of a cocoanut, could be felt, rising out of the pelvis.

*Vaginal Examination.*—Vagina small and elongated. Cervix pressed against pubic bone. Posteriorly, and filling the true pelvis, a firm, smooth, elastic mass connected with the cervix and with the tumor felt above.

*Diagnosis.*—Uterine myoma, partially incarcerated within the pelvis.

Upon October 22d, at the Riverside Hospital, I performed a complete hysterectomy by the supravaginal route and the specimen before us confirms the diagnosis.

In the light of this post-operative knowledge let us consider two points in the clinical history. This patient received electrical treatment from competent hands. You know it is claimed that electricity is particularly effective in dealing with this class of cases and that its use is followed frequently by either disappearance of the growth or of the unpleasant symptoms, or both. Many facts substantiate these claims, but note what happened in this particular case. The patient tells me her physician eight years ago estimated the tumor to be about the size of a lemon. The specimen before you weighs between three and four pounds ; hence, its growth was not checked, though possibly retarded. Some of the symptoms were relieved for a time, but not for long. During the last year her condition has been worse than ever before.

Another general belief is that the menopause cures these tumors as well as all the other ills of woman. Note in this case that the patient's condition has been much worse since the menopause. I would not have you think that the effects of electricity or of the change of life are never curative in uterine myomata, but simply warn you to bear in mind the exceptions as well illustrated by this case, so that you will avoid involving yourselves and your patients in bitter disappointments.

Let us turn to the specimen itself. It is a tumor containing both fibrous and unstriped muscular tissue, hence, its proper name is fibro-myoma; but usually the term fibroma or myoma is applied to such tumors to save ourselves the trouble of using two words. You must remember, however, that such growths never consist of only one kind of tissue. This specimen consists of two lobes, one about the size of a baseball, involving the posterior wall of the uterus; the other, somewhat similar, has its origin in the anterior uterine wall.

The important part of the growth is in the posterior lobe which formed the mass felt lying posterior to the cervix. This division of the tumor when engorged with blood was sufficiently large to become impacted within the true pelvis and give rise to certain pressure symptoms. From your lectures in obstetrics you have become acquainted with the symptoms of that serious condition known as incarceration of the gravid womb. A somewhat similar condition was developing as this tumor, confined within the bony pelvis, slowly increased in size. The bladder, pushed upward, began to show signs of irritation; pressure upon the rectum caused constipation, pressure upon nerve trunks produced pain which radiated into the thighs and, reflexly, disturbed other portions of the nervous system. At the time of operation there was not room sufficient to pass my finger between the tumor and the pelvic wall, and, although there were no adhesions, it required vigorous pulling in order to deliver the tumor from the pelvis. As early as the second day after operation the patient congratulated herself on being free from pain and the discomforts of weight and pressure in the lower part of the abdomen.

In the case of tumors growing upward into the abdomen, a great increase of size is necessary before there are signs of pressure. In the case of the pregnant uterus, we see how readily the abdominal organs adapt themselves to a diminished amount of space. It is very different when the tumor is confined within a bony cavity.

Severe symptoms then develop while the growth is comparatively small, and, following the symptoms due to disturbances of neighboring organs, we may get evidences of gangrene of the tumor itself. The main indication for operation in this case was not the absolute size of the tumor, but its situation. I have seen patients who were quite comfortable with fibroids three times the size of this one, because the growth was upward into the abdomen instead of downward into the pelvis.

You remember the patient complained of profuse leucorrhea and as I lay open the uterine cavity about a tablespoonful of pus escapes. Endometritis is a very common accompaniment of uterine myoma and is frequently of a hemorrhagic form, but in this case there is no history of metrorrhagia. As we examine this specimen we see that the fibro-myomatous growth has been away from and not toward the uterine cavity, whereas, hemorrhage is most common when the tumor grows under the mucous membrane into the cavity of the uterus.

On the other hand, this endometrium, twice the normal size and with a more or less deranged circulation, has afforded a favorable ground for the growth of septic bacteria and this profuse discharge, continuing for several years, has played an important part in producing anemia and loss of strength.

As to the cause of these tumors very little is known. Sterility is given as an etiological factor, but some authors regard sterility as a result instead of a cause. This patient, you remember, has been married twenty years and has never become pregnant. Observation has established the fact that race plays an important part etiologically, the negro race being most frequently affected.

In conclusion, I will sum up as follows :

*First*—This case affords an instance of the growth of a fibromyoma not prevented by the use of electricity nor the establishment of the menopause.

*Second*—The leading symptoms forming the indications for operation were the results of the incarceration of the tumor within the pelvis.

*Third*—The specimen itself illustrates the subserous and interstitial varieties of fibro-myomata with purulent endometritis.

(NOTE.—The patient made a good recovery and was walking about her room three weeks from date of operation.)

